# Anatomical structure of the medullary cavity of proximal femur with three-dimensional computed tomography

**DOI:** 10.1186/s12891-025-08588-x

**Published:** 2025-04-05

**Authors:** Tianhao Shi, Xiaoyang Jia, Kun Zhang, Gengxin Jia, Zhenqi Yang, Minfei Qiang, Yanxi Chen

**Affiliations:** Department of Orthopedic Surgery, Zhongshan Hospital, Fudan University, 180 Fenglin Rd, Shanghai, 200032 China

**Keywords:** Intertrochanteric hip fracture, Lateral wall, Intramedullary nailing, Femoral medullary cavity, Three-dimensional computed tomography (3D-CT)

## Abstract

**Background:**

The lateral femoral wall is an important anatomical parameter of the proximal femur, but intramedullary nail fixation for intertrochanteric fractures may cause iatrogenic lateral wall fractures due to population-based design differences. This study aims to measure the anatomical parameters of the proximal femoral medullary cavity and provide data to help design intramedullary nails tailored to the Chinese population to reduce the risk of complications such as lateral wall fractures.

**Methods:**

Consecutive patients undergoing full-length or upper half CT scans of the femur were included from January 2010 to December 2021. The anatomical parameters of medullary cavity were defined and measured, including prominence length, canal-shaft angle and proximal minimum diameter. Intraclass Correlation Coefficient (ICC) was used to estimate the inter- and intra-observer agreements.

**Results:**

A total of 168 patients, comprising 78 men and 90 women, were included. The mean prominence length was 67.4 ± 4.9 mm (males: 70.8 ± 3.6 mm, females: 64.4 ± 3.9 mm). The mean canal-shaft angle was 5.5° ± 0.7° (males: 5.6 ± 0.8°, females: 5.5 ± 0.7°). The mean proximal minimum diameter was 22.7 ± 1.8 mm (males: 24.0 ± 1.5 mm, females: 21.6 ± 1.4 mm) at the level of 1/3 prominence length from bottom to top. Gender differences were observed in these parameters (*p* < 0.001) except for the canal-shaft angle (*p* = 0.45). The mean proximal minimum diameter was significantly larger in the group aged 50 years and older (23.1 ± 1.7 mm) compared to the group younger than 50 years (22.4 ± 1.9 mm) (*p* = 0.02). Inter- and intra-observer agreement was almost perfect for all the parameters (all ICC values > 0.8).

**Conclusions:**

Males have a longer prominence length and larger proximal minimum diameter than females. The proximal minimum diameter is larger in the older population than in the younger population. The measurement results help support the design of intramedullary nails tailored to the Chinese population.

**Clinical trial number:**

Not applicable.

## Introduction

Hip fractures are the most prevalent type of fractures in the elderly population, disabling 4.5 million people worldwide every year [1, 2]. As the population ages, the incidence of these fractures continues to rise, with nearly half being intertrochanteric fractures [3]. When not treated promptly, these fractures result in poor outcomes [4]. Surgical intervention is the primary approach for treating intertrochanteric fractures and intramedullary nails are widely used due to their biomechanical advantages [5]. Research shows that intramedullary nails can lead to iatrogenic lateral wall fractures [6–8]. The integrity of the lateral wall is vital for ensuring internal fixation stability in intertrochanteric fractures, which can affect postoperative function [9]. It has been identified as an important predictor of a reoperation in unstable intertrochanteric fractures [10]. Iatrogenic lateral wall fractures may result from improper nail insertion paths or forceful insertion during surgery, as well as the weak state of lateral wall [11]. Apart from these factors, the risk of iatrogenic lateral wall fractures may also relate to the design of intramedullary nails. Mainstream intramedullary nails are designed derived from the femoral anatomy of Western populations. Compared to Western populations, Asians typically have narrower medullary canals, shorter femoral necks, and smaller neck-shaft angles [12–14]. These anatomical differences between Asian and Western populations contribute to a mismatch between intramedullary nails and the proximal femoral structure in Asians. The narrower medullary canal in Asians increases the risk of the intramedullary nail compressing the lateral cortex, leading to lateral wall fractures. Even the widely used Asian-modified PFNA-II (Proximal Femoral Nail Antirotation-II) can cause iatrogenic fractures [6]. Studies also reported its proximal nail protrusion and distal nail impingement in Chinese populations, indicating the need for further design improvements [15–17].

Currently, there is no intramedullary fixation system designed specifically for Chinese individuals. Despite large-scale studies measuring femoral parameters in Asians [18–20], sufficient data on the anatomical parameters of the proximal femoral medullary cavity in the Chinese population is still lacking. Therefore, this study aims to use three-dimensional reconstruction models of the femur to measure some important anatomical parameters related to the proximal femoral medullary cavity in the Chinese population, which are directly associated with the matching of intramedullary nails. The significance of this study lies in designing intramedullary nails anatomically suitable for the Chinese population based on these anatomical parameters.

## Materials and methods

### Study population

Consecutive patients aged 18 years and above who had full-length or upper half CT scans of the femur were included from January 1, 2010 to December 31, 2021. These patients primarily included trauma patients (who were later diagnosed with soft tissue injuries or milder conditions, rather than fractures) and patients who underwent lower limb CT angiography due to vascular-related conditions. The exclusion criteria included hip osteoarthritis, femoral fractures, previous hip surgeries, femoral deformities, or any history of femoral trauma. Hospital information and medical imaging systems provided demographic and imaging information of patients across outpatient and inpatient settings. A young orthopedic surgeon reviewed the clinical and imaging data to confirm eligibility. This study was approved by the Institutional Review Board of East Hospital, Tongji University School of Medicine, which also waived the requirement for informed consent due to the use of deidentified data.

### Three-dimensional model reconstruction

A series of CT axial images of the proximal femur were acquired using a 16- or 32-detector spiral CT scanner (GE LightSpeed; GE Medical Systems) and then imported into the computer-assisted orthopedic clinical research platform (SuperImage system, orthopedic edition 1.1; Cybermed) [21–23]. Three-Dimensional models of the proximal femur were reconstructed at 0.625 mm interval by a surface shading algorithm. Different bones were labeled and unrelated bones were removed by using an interactive automatic segmentation technique. Cortical bone and medullary cavity were differentiated based on CT thresholds. 77.

### Parameter measurements

**Fig. 1:**
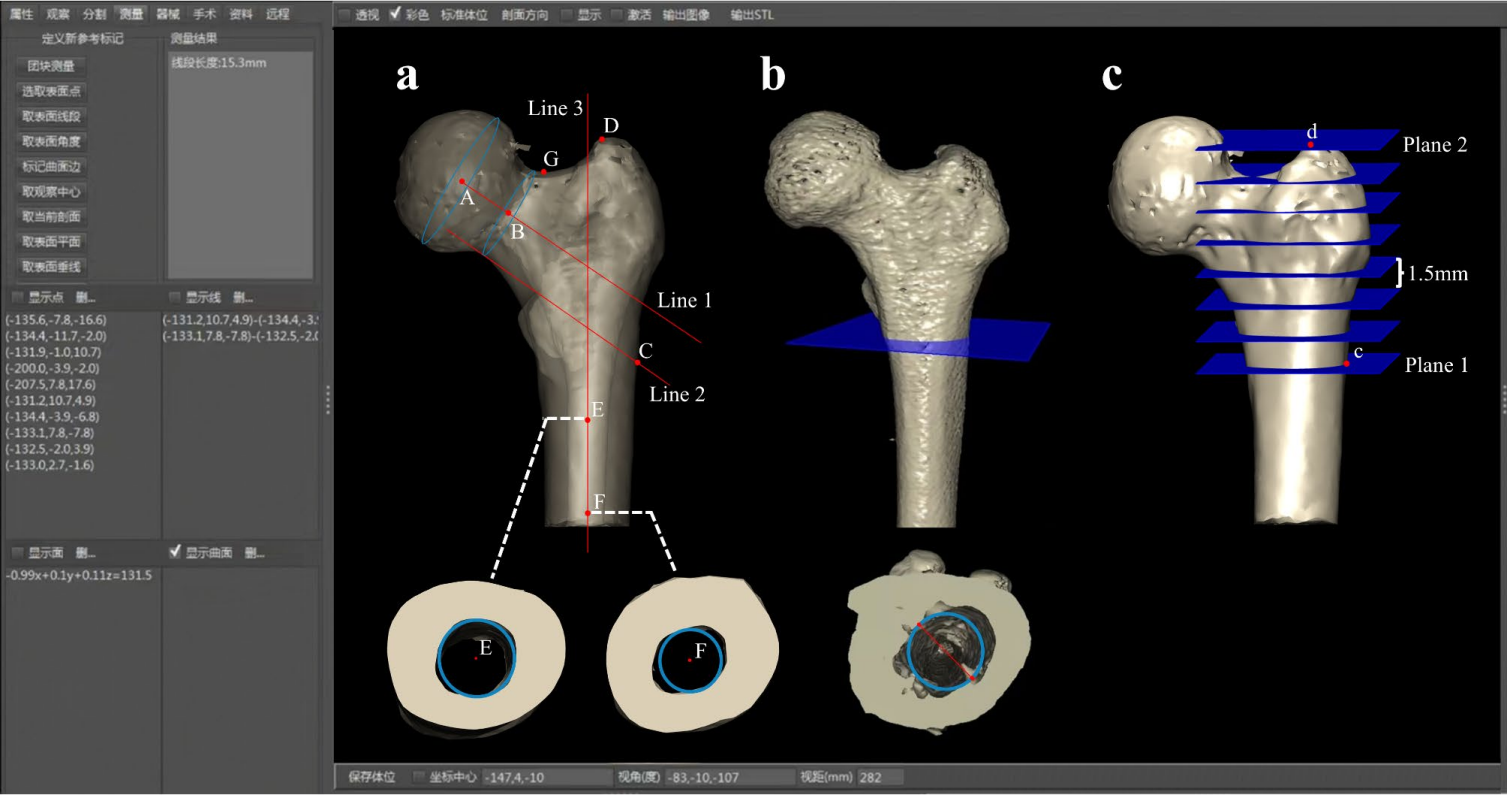
Relevant points, lines, and planes were defined. (**a**) Point A and B were the centers of femoral head and femoral neck, respectively. Line 1 was the axis of the femoral neck formed by point A and point B. Line 2 was parallel to line 1. It passed through the inferior margin of the femoral neck and intersected the lateral cortex at point C. Point D was the greater trochanter apex. Points E and F were the centers of the best fit circles within the medullary cavity at approximately 2 cm and 5 cm below the lesser trochanter, respectively. Line 3 was the femoral shaft axis formed by point E and point F. Point G was the lowest point on the superior margin of the femoral neck; (**b**) The best fit circle, its center, as well as the minimum diameter, were automatically generated by the computer-assisted orthopedic clinical research platform (SuperImage system, orthopedic edition 1.1; Cybermed); (**c**) A series of planes were created at 1.5 mm intervals from plane 1 (the level of point c) to plane 2 (the level of point d), dividing the proximal femur into several slices

**Fig. 2:**
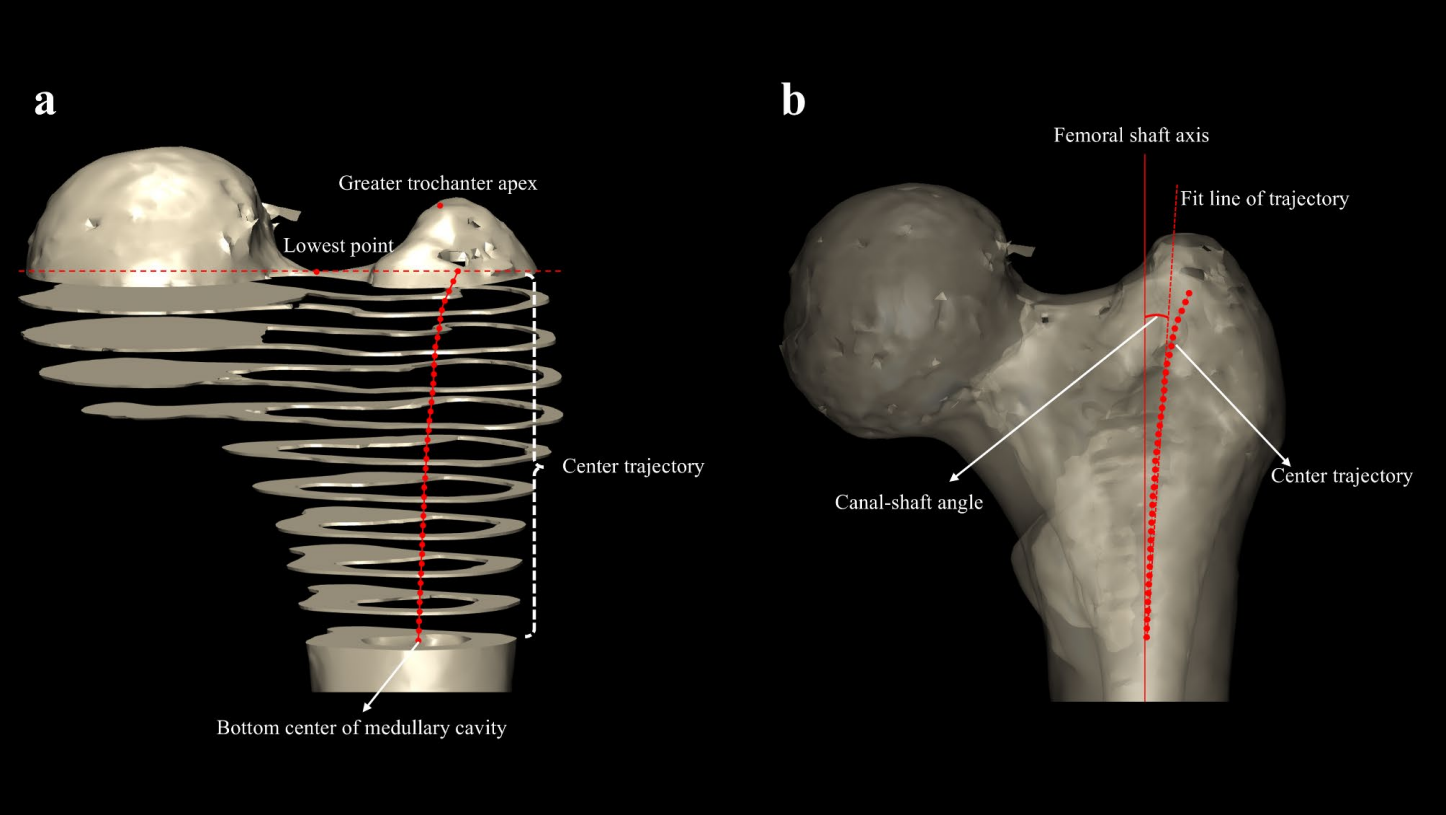
(**a**) Best fit circles were drawn for the medullary cavity in each slice from bottom to top, and the center of these circles were connected to form a trajectory. Considering that the region near the greater trochanter apex would inevitably be destroyed during surgery, the area above the lowest point on the superior margin of the femoral neck was excluded; (**b**) A fit line was drawn along the center trajectory, and it passed through the bottom center of medullary cavity. The canal-shaft angle was defined as the angle between the fit line and the femoral shaft axis in the anterior view

Important reference points, lines, and planes were defined as follows for further measurement of the proximal femoral medullary cavity. The lower boundary was the horizontal plane at the intersection (Point C) of the lateral cortex and the parallel line to the axis of the femoral neck which passed through the inferior margin of the femoral neck. The upper boundary was the horizontal plane that passed through the greater trochanter apex (Point D) (Fig. 1a). Best fit circles and their centers for the medullary cavity were automatically generated by our software in each slice from bottom to top after manually selecting the contour of medullary cavity (Fig. 1b). A series of planes were created at 1.5 mm intervals from the lower bottom boundary (Plane 1) to the upper boundary (Plane 2), dividing the proximal femur into several slices (Fig. 1c). After obtaining a series of planes, a series of fitted circle centers for the medullary cavity were generated in each plane. A trajectory was formed by these centers. Due to the irregular shape of the medullary cavity near the greater trochanter apex, the generated centers in this region deviated significantly from the actual path of the intramedullary nail. Additionally, the region is inevitably destroyed during nail insertion in surgery; therefore, the area above the lowest point on the superior margin of the femoral neck was excluded (Fig. 2a). A fit line was drawn along the center trajectory, passing through the bottom center of medullary cavity, based on the coordinates of each center. The canal-shaft angle was determined by the fit line of the trajectory and the femoral shaft axis in the anterior view, which was used to assess the proper medial-lateral angel of intramedullary nails (Fig. 2b).

**Fig. 3:**
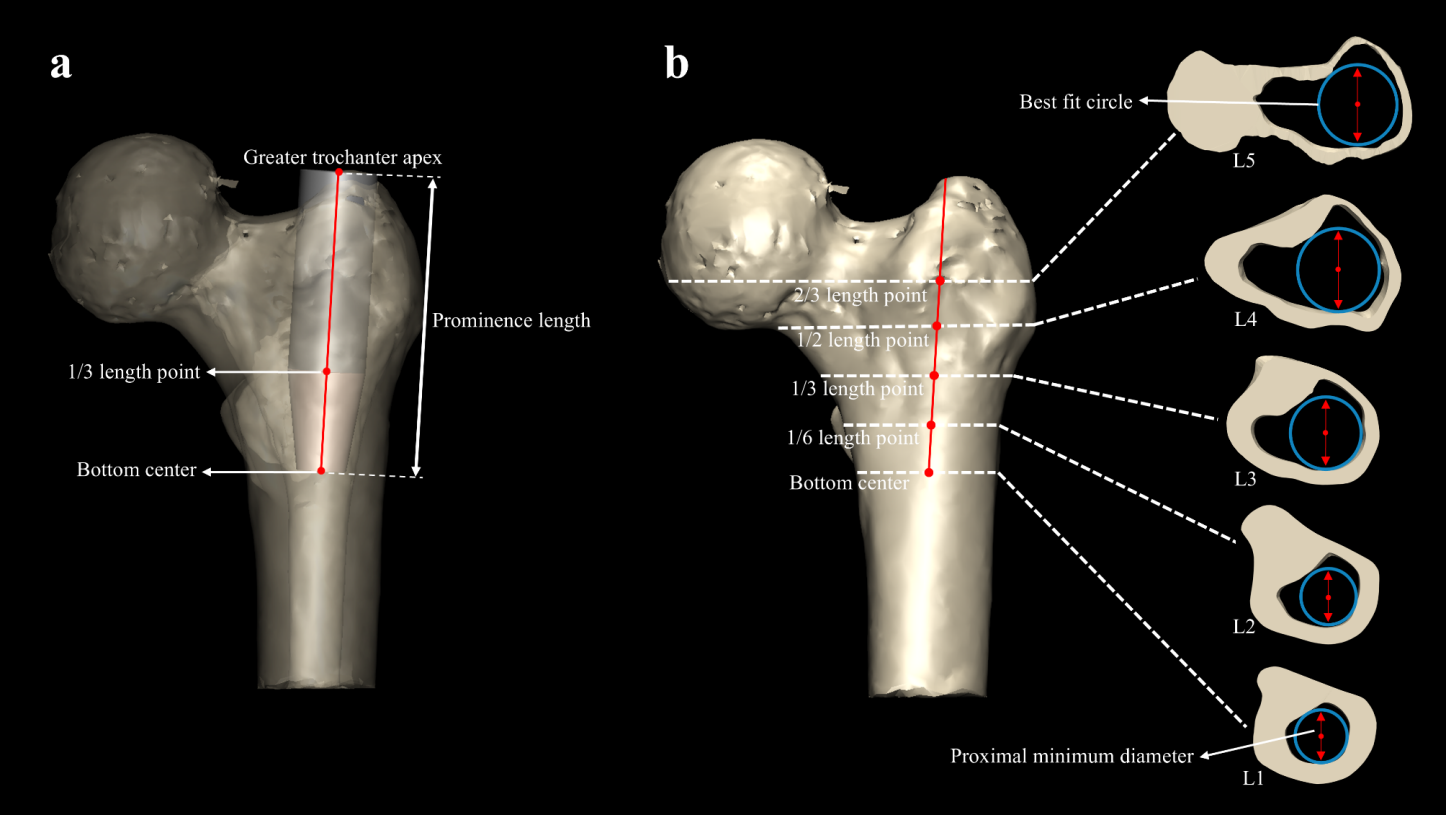
(**a**) The prominence length was defined as the distance between the great trochanter apex and the bottom center of medullary cavity to describe the length of the proximal portion of intramedullary nail. The simulated proximal intramedullary nail was combined by the teardrop-shaped lower one-third (brown part) and the cylindrical upper two-thirds (grey part); (**b**) The slice and proximal minimum diameter were shown at five levels from bottom to top, corresponding to the bottom center, 1/6 of the prominence length, 1/3 of the prominence length, 1/2 of the prominence length and 2/3 of the prominence length

The prominence length was defined as the distance between the great trochanter apex and the bottom center of medullary cavity to describe the length of the proximal portion of intramedullary nail (Fig. 3a). The proximal minimum diameter was automatically generated by the software based on its center (Fig. 1b). It was the diameter of the smallest circle that just tangentially meet the inner side of the cortex. Although the proximal minimum diameter of all slices was measured, five additional levels of the proximal minimum diameter were added based on specific proportions of the prominence length for better comparison from bottom to top, corresponding to the bottom center, 1/6 of the prominence length, 1/3 of the prominence length, 1/2 of the prominence length and 2/3 of the prominence length (Fig. 3b).

### Statistical analysis

Normality of all parameters was assessed with the Kolmogorov-Smirnov test. Quantitative variables were presented as means with standard deviations (SD) or medians with interquartile ranges. Gender and age differences were analyzed using an independent-sample *t*-test or Mann–Whitney *U-*test. A two-sided *p* value below 0.05 was considered statistically significant. The Intraclass Correlation Coefficient (ICC) for the two-way random effects model was used to estimate the inter- and intra-observer agreements. ICC values ranged from 0 to 1, with interpretations as follows: ICC < 0.2 indicated poor agreement, 0.2 to 0.4 indicated fair agreement, 0.4 to 0.6 indicated moderate agreement, 0.6 to 0.8 indicated substantial agreement, and 0.8 to 1.0 indicated almost perfect agreement. Data analysis and plotting were performed by SPSS software (version 26.0, IBM Corporation, Armonk, NY, USA).

## Results

Among these patients, there were 78 (46.4%) men (mean age, 46.2 ± 9.5 years; range, 21 to 74 years) and 90 (53.6%) women (mean age, 49.4 ± 10.0 years; range, 21 to 75 years).

**Table 1:** Anatomical parameters of the medullary cavity of proximal femur

Parameters	Total(*n* = 168)	Male(*n* = 78)	Female(*n* = 90)	*P* ^*^
**Prominence length (mm)**	67.4 ± 4.9	70.8 ± 3.6	64.4 ± 3.9	< 0.001
**Canal-shaft angle (°)**	5.5 ± 0.7	5.6 ± 0.8	5.5 ± 0.7	0.45
**Proximal minimum diameter**^**#**^ (**mm**)				
L1 (bottom)	17.8 ± 2.1	18.8 ± 2.1	16.8 ± 1.5	< 0.001
L2 (1/6)	19.8 ± 1.9	20.9 ± 1.8	18.8 ± 1.4	< 0.001
L3 (1/3)	22.7 ± 1.8	24.0 ± 1.5	21.6 ± 1.4	< 0.001
L4 (1/2)	27.0 ± 2.0	28.4 ± 1.4	25.8 ± 1.5	< 0.001
L5 (2/3)	24.1 ± 2.2	25.5 ± 1.9	22.9 ± 1.6	< 0.001

The results all follow a normal distribution so they are expressed as mean ± standard deviation

^*^P values are based on independent-sample t-test

^**#**^Starting from bottom to top, levels (L1-L5) were measured at intervals of 1/6 of the prominence length up to 2/3 of its total length

### Prominence length

**Fig. 4:**
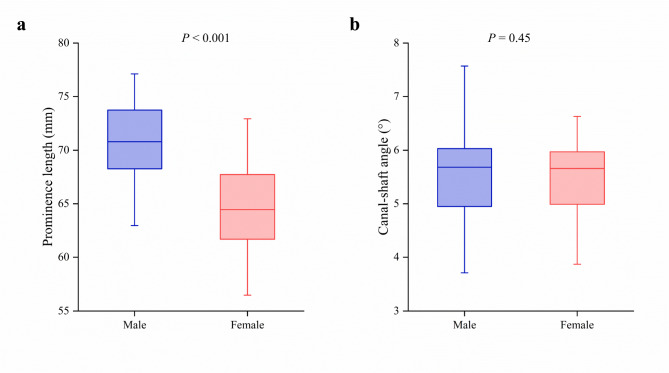
Gender differences in prominence length and canal-shaft angle. (**a**) Prominence length; (**b**) Canal-shaft angle

The mean prominence length was 67.4 ± 4.9 mm (range, 56.5 to 77.1 mm). The average prominence length in males was about 6 to 7 mm longer than in females (Table 1; Fig. 4a, *p* < 0.001).

### Canal-shaft angle

The mean canal-shaft angle was 5.5° ± 0.7° (range, 3.7° to 7.9°). The canal-shaft angle was similar between males and females, with no significant gender differences observed (Table 1; Fig. 4b, *p* = 0.45).

### Proximal minimum diameter

**Fig. 5:**
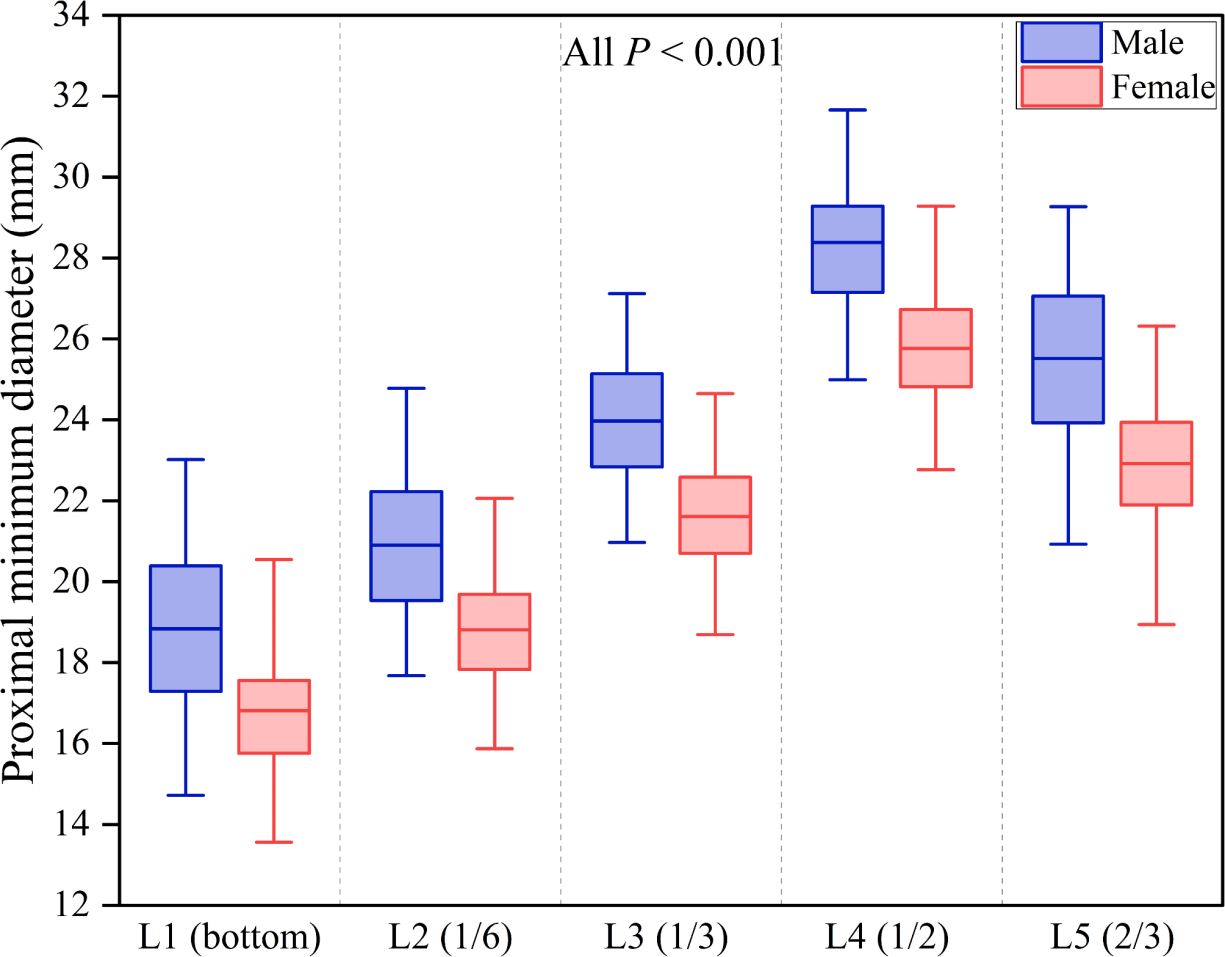
Gender differences in proximal minimum diameter at five levels. The five levels were obtained from bottom to top, corresponding to the bottom, 1/6 of the prominence length, 1/3 of the prominence length, 1/2 of the prominence length and 2/3 of the prominence length

The proximal minimum diameter was compared across five representative levels. The mean proximal minimum diameter from L1 to L5 was 17.8 ± 2.1 mm (range, 13.6 to 23.0 mm), 19.8 ± 1.9 mm (range, 15.9 to 24.8 mm), 22.7 ± 1.8 mm (range, 18.7 to 27.1 mm), 27.0 ± 2.0 mm (range, 22.8 to 31.7 mm) and 24.1 ± 2.2 mm (range, 18.9 to 29.3 mm), respectively. The results indicated a trend of widening followed by narrowing from bottom to top. Besides, the average proximal minimum diameter in males was greater than in females at all the five levels (Table 1; Fig. 5, all *p* < 0.001).

### Age-based analysis

Based on the characteristics of osteoporosis onset [24, 25], the population was divided into two groups: individuals younger than 50 years and those aged 50 years or older. The mean proximal minimum diameter for individuals aged 50 and older (*n* = 75) was 23.1 ± 1.7 mm, while for those younger than 50 (*n* = 93), it was 22.4 ± 1.9 mm (*p* = 0.02). Within each age group, comparisons were made between males and females. In women, the mean proximal minimum diameter for those aged 50 and older (*n* = 49) was 22.3 ± 1.2 mm, while for those younger than 50(*n* = 41), it was 20.8 ± 1.0 mm (*p* < 0.001). In men, the mean proximal minimum diameter for those aged 50 and older (*n* = 26) was 24.6 ± 1.4 mm, while for those younger than 50 (*n* = 52), it was 23.7 ± 1.4 mm (*p* = 0.006). The results showed that the mean proximal minimum diameter was significantly larger in the group aged 50 years and older compared to the group younger than 50 years, with this difference being observed both overall and within each gender subgroup.

### Inter- and intra-observer reliability

The intra-observer agreement for prominence length, canal-shaft angle, and proximal minimum was 0.975, 0.930 and 0.958, respectively. The inter-observer agreement for prominence length, canal-shaft angle, and proximal minimum was 0.932, 0.879 and 0.914, respectively. Inter- and intra-observer agreement was almost perfect for all the anatomical parameters (all ICC values > 0.8).

## Discussion

This study used 3D reconstruction models of the proximal femur to measure important anatomical parameters in Chinese individuals. Based on these anatomical parameters, it would be possible to further design proximal femoral intramedullary nails anatomically suited to the Chinese population. Currently, mainstream intramedullary nails designed based on Western anatomical parameters do not fit well with Asian populations [14]. However, the anatomical structure of the Chinese population remains under-researched. There are some previous measurement made by cadavers, radiographs or conventional CT [26–29]. However, these studies are limited by small sample sizes and the lack of more precise measurements through three-dimensional reconstruction. This study used 168 cases of 3D reconstruction models to ensure measurement accuracy and representativeness. And the ICC values indicated almost perfect inter- and intra-observer agreements.

The definition of prominence length is intended to explore the appropriate length of the proximal portion of the intramedullary nail. Designing an appropriate prominence length is critical for surgical success and patient comfort. The result showed the mean prominence length was 67.4 mm. Excessive length at the proximal end may cause the intramedullary nail to protrude beyond the greater trochanter. Attempting to insert this protruding part forcefully can lead to rupture of the lateral wall during surgery. The protruding proximal end may also induce hip pain in patients postoperatively [15, 30]. On the other hand, If the length is too short, it may cause difficulty in removing the nail.

The proximal minimum diameter acts as a reference for the proximal diameter of intramedullary nail. Experts recommended that the reaming diameter should be 1–2 mm larger than the diameter of the nail [18, 31]. The proximal diameter of the nail should be slightly smaller than the proximal minimum diameter to provide adequate mechanical support without compressing the lateral wall. Our complete result measured from bottom to top at 1.5 mm intervals was not shown due to the large number of slices. Instead, the minimum diameter was measured additionally at five levels based on specific proportions of the prominence length to get a clear comparison across different regions. The results indicated a trend of widening followed by narrowing for the medullary Cavity from bottom to top. In the lower third part (L1 to L3), the geometry of the medullary cavity is approximately teardrop-shaped. Designing the intramedullary nail with a teardrop shape for this part may better match the medullary cavity. Besides, age is an important factor influencing the diameter of the medullary cavity, with older individuals having a wider medullary cavity. Additionally, the effect of age on the medullary cavity diameter is more evident in women than in men, likely due to the higher rate of bone loss in women. This also explains why women in the elderly population are more prone to intertrochanteric femoral fractures compared to men.

Both prominence length and proximal minimum diameter exhibit significant gender differences (*p* < 0.001). To better match different femoral anatomical structure, we recommend designing the proximal length and diameter of intramedullary nails to be variable, with options available for both males and females.

The canal-shaft angle was compared to the medial-lateral angle of intramedullary nails, which plays an important role in determining the nail insertion path together with the entry point. An incorrect angle may cause difficulties during insertion or lead to complications such as fracture of the inner or outer cortical bone due to compression. To obtain a suitable insertion path, previous studies suggested placing the entry point 5 mm posterior, 5 mm medial, or 5 mm posteromedial to the greater trochanter apex [32, 33]. The medial-lateral angle was also reduced from 6° in the PFNA to 5° in the PFNA-II to better fit the Asian population [34]. Our study results showed no significant gender differences in the canal-shaft angle, which had a mean canal-shaft angle of 5.5° and was close to the angle of the PFNA and PFNA-II.

Some other research also measured parameters that were similar to those defined in our study. Hu et al. measured the length of the protruding part of the PFNA-II, suggesting that the PFNA-II need to shorten the proximal nail end 5 to 10 mm [15]. However, they did not directly measure the prominence length. A study in India found the mean prominence length in Indian populations to be 65.73 mm and the mean trochanteric shaft angle to be 10.45° [27]. Another Korean study found the mean prominence length was 61.1 mm, but their endpoint of the length was defined above the lesser tuberosity. And their mean medial-lateral angle was 8.4° [26]. Our prominence length is comparable to their results, but our angle is significantly smaller than theirs, which may be due to inconsistent definitions and varying measurement methods. Their medial-lateral angle was defined by drawing a line from the greater trochanter apex to its intersection with the femoral shaft axis at a certain height on the coronal plane. In contrast, our definition took into account the trajectory changes of the medullary cavity center based on 3D models and fit this curved trajectory into a straight line, which might better reflect changes in the shape of the medullary cavity.

This study has some limitations that shall be acknowledged. First, this study did not account for potential variations such as body height and behavioral factors, which could influence femoral structure. Body height is a critical factor in bone anatomy, as it significantly influences measurements such as prominence length and diameters. Taller individuals tend to have larger bone dimensions [18, 35]. Since males tend to be taller on average than females, it is still unclear whether the observed gender differences in anatomical parameters in our study are mainly due to body height or if they are influenced by hormonal levels and anatomical differences between genders. This issue needs further investigation. Second, this study focuses on the proximal rather than distal femur, without investigating potential issues related to the design of intramedullary nails at the distal end and their compatibility with the curvature of the femoral shaft. Third, this study only measured anatomical parameters and did not provide direct detailed design for intramedullary nails. Future research should establish standardized baselines, include a broader population, and design well-matched intramedullary nails for further fitting tests [21, 22].

## Conclusions

This study used three-dimensional models to investigate the anatomical parameters of the proximal femoral medullary cavity in Chinese individuals. Males have a longer prominence length and larger proximal minimum diameter than females. The proximal minimum diameter is larger in the older population than in the younger population. These findings highlight the importance of developing femoral intramedullary nails specifically tailored for Chinese populations to reduce the incidence of iatrogenic lateral wall fractures and other complications.

## Data Availability

The datasets used and/or analysed during the current study are available from the corresponding author on reasonable request.

## References

[1] Bhandari M, Swiontkowski M. Management of acute hip fracture. N Engl J Med. 2017;377(21):2053–62. 10.1056/NEJMcp1611090 29166235

[2] Burge R, Dawson-Hughes B, Solomon DH, Wong JB, King A, Tosteson A. Incidence and economic burden of osteoporosis-related fractures in the United States, 2005–2025. J Bone Min Res. 2007;22(3):465–75. 10.1359/jbmr.061113 17144789

[3] Dhanwal DK, Dennison EM, Harvey NC, Cooper C. Epidemiology of hip fracture: worldwide geographic variation. Indian J Orthop. 2011;45(1):15–22. 21221218 10.4103/0019-5413.73656 PMC3004072

[4] Baghdadi S, Kiyani M, Kalantar SH, Shiri S, Sohrabi O, Beheshti Fard S, Afzal S, Khabiri SS. Mortality following proximal femoral fractures in elderly patients: a large retrospective cohort study of incidence and risk factors. BMC Musculoskelet Disord. 2023;24(1):693. 37649030 10.1186/s12891-023-06825-9 PMC10466793

[5] Bohl DD, Basques BA, Golinvaux NS, Miller CP, Baumgaertner MR, Grauer JN. Extramedullary compared with intramedullary implants for intertrochanteric hip fractures: thirty-day outcomes of 4432 procedures from the ACS NSQIP database. J Bone Joint Surg Am. 2014;96(22):1871–7. 25410504 10.2106/JBJS.N.00041

[6] Jia X, Qiang M, Zhang K, Han Q, Jia G, Shi T, Wu Y, Chen Y. Accuracy of detecting burst of the lateral wall in intertrochanteric hip fractures with plain radiographs: is postoperative CT necessary? Heliyon. 2024;10(3):e25389. 10.1016/j.heliyon.2024.e25389 PMC10865257 38356592

[7] Zhang S, Zhang K, Wang Y, Feng W, Wang B, Yu B. Using three-dimensional computational modeling to compare the geometrical fitness of two kinds of proximal femoral intramedullary nail for Chinese femur. ScientificWorldJournal. 2013;2013:978485. 10.1155/2013/978485 PMC3575634 23431263

[8] Hwang JH, Oh JK, Han SH, Shon WY, Oh CW. Mismatch between PFNa and medullary canal causing difficulty in nailing of the pertrochanteric fractures. Arch Orthop Trauma Surg. 2008;128(12):1443–6. 18784928 10.1007/s00402-008-0736-1

[9] Hao Y, Zhang Z, Zhou F, Ji H, Tian Y, Guo Y, Lv Y, Yang Z, Hou G. Risk factors for implant failure in reverse oblique and transverse intertrochanteric fractures treated with proximal femoral nail antirotation (PFNA). J Orthop Surg Res. 2019;14(1):350. 31703710 10.1186/s13018-019-1414-4 PMC6842253

[10] Palm H, Jacobsen S, Sonne-Holm S, Gebuhr P, Hip Fracture Study G. Integrity of the lateral femoral wall in intertrochanteric hip fractures: an important predictor of a reoperation. J Bone Joint Surg Am. 2007;89(3):470–5. 17332094 10.2106/JBJS.F.00679

[11] Hsu CE, Shih CM, Wang CC, Huang KC. Lateral femoral wall thickness. A reliable predictor of post-operative lateral wall fracture in intertrochanteric fractures. Bone Joint J. 2013;95–B(8):1134–8. 23908432 10.1302/0301-620X.95B8.31495

[12] Cummings SR, Cauley JA, Palermo L, Ross PD, Wasnich RD, Black D, Faulkner KG. Racial differences in hip axis lengths might explain racial differences in rates of hip fracture. Study of osteoporotic fractures research group. Osteoporos Int. 1994;4(4):226–9. 7949753 10.1007/BF01623243

[13] Chin K, Evans MC, Cornish J, Cundy T, Reid IR. Differences in hip axis and femoral neck length in premenopausal women of polynesian, Asian and European origin. Osteoporos Int. 1997;7(4):344–7. 9373568 10.1007/BF01623775

[14] Schmutz B, Kmiec S Jr., Wullschleger ME, Altmann M, Schuetz M. 3D computer graphical anatomy study of the femur: a basis for a new nail design. Arch Orthop Trauma Surg. 2017;137(3):321–31. 28168640 10.1007/s00402-016-2621-7

[15] Hu SJ, Chang SM, Ma Z, Du SC, Xiong LP, Wang X. PFNA-II protrusion over the greater trochanter in the Asian population used in proximal femoral fractures. Indian J Orthop. 2016;50(6):641–6. 27904220 10.4103/0019-5413.193475 PMC5122260

[16] Chang SM, Song DL, Ma Z, Tao YL, Chen WL, Zhang LZ, Wang X. Mismatch of the short straight cephalomedullary nail (PFNA-II) with the anterior bow of the femur in an Asian population. J Orthop Trauma. 2014;28(1):17–22. 24121985 10.1097/BOT.0000000000000022

[17] Mukherjee K, Prashanth KRT, M RT, Kumar RD. Mismatch of short straight proximal femur nails with anterior bow of femur in Indian population- a radiological and functional analysis. J Orthop. 2022;29:65–70. 35145329 10.1016/j.jor.2022.01.006 PMC8814591

[18] Thiesen DM, Prange F, Berger-Groch J, Ntalos D, Petersik A, Hofstatter B, Rueger JM, Klatte TO, Hartel MJ. Femoral antecurvation-A 3D CT analysis of 1232 adult femurs. PLoS ONE. 2018;13(10):e0204961. 30300421 10.1371/journal.pone.0204961 PMC6177158

[19] Maratt J, Schilling PL, Holcombe S, Dougherty R, Murphy R, Wang SC, Goulet JA. Variation in the femoral bow: a novel high-throughput analysis of 3922 femurs on cross-sectional imaging. J Orthop Trauma. 2014;28(1):6–9. 23799352 10.1097/BOT.0b013e31829ff3c9

[20] Wuestemann T, Hoare SG, Petersik A, Hofstaetter B, Fehily M, Matsubara M, Markel DC. Bone morphology of the proximal femoral canal: ethnicity related differences and the influence on cementless tapered wedge stem designs. Hip Int. 2021;31(4):482–91. 31868035 10.1177/1120700019895458

[21] Jia X, Zhang K, Qiang M, Wu Y, Chen Y. Association of computer-assisted virtual preoperative planning with postoperative mortality and complications in older patients with intertrochanteric hip fracture. JAMA Netw Open. 2020;3(8):e205830. 32777058 10.1001/jamanetworkopen.2020.5830 PMC7417968

[22] Jia X, Zhang K, Qiang M, Han Q, Zhao G, Wu Y, Chen Y. Design of well-matched end-structure of anatomical proximal femoral locking plate based on computer-assisted imaging combined with 3D printing technology: a quality improvement study. Int J Surg. 2023;109(5):1169–79. 37026794 10.1097/JS9.0000000000000300 PMC10389635

[23] Chen Y, Jia X, Qiang M, Zhang K, Chen S. Computer-assisted virtual surgical technology versus three-dimensional printing technology in preoperative planning for displaced three and four-part fractures of the proximal end of the humerus. J Bone Joint Surg Am. 2018;100(22):1960–8. 30480600 10.2106/JBJS.18.00477

[24] Wang L, Yu W, Yin X, Cui L, Tang S, Jiang N, Cui L, Zhao N, Lin Q, Chen L, et al. Prevalence of osteoporosis and fracture in China: the China osteoporosis prevalence study. JAMA Netw Open. 2021;4(8):e2121106. 34398202 10.1001/jamanetworkopen.2021.21106 PMC8369359

[25] Zeng Q, Li N, Wang Q, Feng J, Sun D, Zhang Q, Huang J, Wen Q, Hu R, Wang L, et al. The prevalence of osteoporosis in China, a nationwide, multicenter DXA survey. J Bone Min Res. 2019;34(10):1789–97. 10.1002/jbmr.3757 31067339

[26] Tyagi V, Yang JH, Oh KJ. A computed tomography-based analysis of proximal femoral geometry for lateral impingement with two types of proximal femoral nail anterotation in subtrochanteric fractures. Injury. 2010;41(8):857–61. 20537641 10.1016/j.injury.2010.04.018

[27] Pathrot D, Ul Haq R, Aggarwal AN, Nagar M, Bhatt S. Assessment of the geometry of proximal femur for short cephalomedullary nail placement: an observational study in dry femora and living subjects. Indian J Orthop. 2016;50(3):269–76. 27293287 10.4103/0019-5413.181785 PMC4885295

[28] Kulkarni M, Naik AM, Shetty CB, Paruthikunnan SM, Rao SK. CT based measurement of anatomical dimensions of femur and its relevance in nail designs for proximal femoral fractures. J Orthop. 2020;20:63–9. 32042232 10.1016/j.jor.2019.12.002 PMC7000424

[29] Zhao R, Cai H, Tian H, Zhang K. CT measurements of the proximal femoral medullary cavity in healthy adults: a cross-sectional study. J Pak Med Assoc. 2023;73(12):2363–9. 38083913 10.47391/JPMA.7538

[30] Kim SS, Kim HJ, Lee CS. Clinical outcomes of PFNA-II in the Asian intertrochanteric fracture patients: comparison of clinical results according to proximal nail protrusion. Injury. 2020;51(2):361–6. 31812322 10.1016/j.injury.2019.11.040

[31] Haidukewych GJ. Intertrochanteric fractures: ten tips to improve results. J Bone Joint Surg Am. 2009;91(3):712–9. 19255235

[32] Farhang K, Desai R, Wilber JH, Cooperman DR, Liu RW. An anatomical study of the entry point in the greater trochanter for intramedullary nailing. Bone Joint J. 2014;96–B(9):1274–81. 25183603 10.1302/0301-620X.96B9.34314

[33] Pan S, Liu XH, Feng T, Kang HJ, Tian ZG, Lou CG. Influence of different great trochanteric entry points on the outcome of intertrochanteric fractures: a retrospective cohort study. BMC Musculoskelet Disord. 2017;18(1):107. 28288607 10.1186/s12891-017-1472-x PMC5348905

[34] Sawaguchi T, Sakagoshi D, Shima Y, Ito T, Goldhahn S. Do design adaptations of a trochanteric nail make sense for Asian patients? Results of a multicenter study of the PFNA-II in Japan. Injury. 2014;45(10):1624–31. 24985469 10.1016/j.injury.2014.06.002

[35] Rubin PJ, Leyvraz PF, Aubaniac JM, Argenson JN, Esteve P, de Roguin B. The morphology of the proximal femur. A three-dimensional radiographic analysis. J Bone Joint Surg Br. 1992;74(1):28–32. 1732260 10.1302/0301-620X.74B1.1732260

